# Placental pathology and neonatal morbidity: exploring the impact of gestational age at birth

**DOI:** 10.1186/s12884-024-06392-4

**Published:** 2024-03-14

**Authors:** Elisabeth B. Budal, Jørg Kessler, Geir Egil Eide, Cathrine Ebbing, Karin Collett

**Affiliations:** 1 Department of Clinical Medicine, University of Bergen, Bergen, Norway; 2 Department of Pathology, Haukeland University Hospital, Bergen, Norway; 3 Department of Obstetrics and Gynecology, Haukeland University Hospital, Bergen, Norway; 4 Centre for Clinical Research, Haukeland University Hospital, Bergen, Norway; 5 Department of Global Public Health and Primary Care, University of Bergen, Bergen, Norway; 6 Western Norway University of Applied Sciences, Bergen, Norway; 7 Department of Pathology, Helse Bergen HF, Haukeland University Hospital, Post box 1400, Bergen, N-5021 Norway

**Keywords:** Term neonates, Placental pathology, Clinical characteristics, Perinatal outcome

## Abstract

**Aim:**

To evaluate placental pathology in term and post-term births, investigate differences in clinical characteristics, and assess the risk of adverse neonatal outcome.

**Methods:**

This prospective observational study included 315 singleton births with gestational age (GA) > 36 weeks + 6 days meeting the local criteria for referral to placental histopathologic examination. We applied the Amsterdam criteria to classify the placentas. Births were categorized according to GA; early-term (37 weeks + 0 days to 38 weeks + 6 days), term (39 weeks + 0 days to 40 weeks + 6 days), late-term (41 weeks + 0 days to 41 weeks + 6 days), and post-term births (≥ 42 weeks + 0 days). The groups were compared regarding placental pathology findings and clinical characteristics. Adverse neonatal outcomes were defined as 5-minute Apgar score < 7, umbilical cord artery pH < 7.0, admission to the neonatal intensive care unit or intrauterine death. A composite adverse outcome included one or more adverse outcomes. The associations between placental pathology, adverse neonatal outcomes, maternal and pregnancy characteristics were evaluated by logistic regression analysis.

**Results:**

Late-term and post-term births exhibited significantly higher rates of histologic chorioamnionitis (HCA), fetal inflammatory response, clinical chorioamnionitis (CCA) and transfer to neonatal intensive care unit (NICU) compared to early-term and term births. HCA and maternal smoking in pregnancy were associated with adverse outcomes in an adjusted analysis. Nulliparity, CCA, emergency section and increasing GA were all significantly associated with HCA.

**Conclusions:**

HCA was more prevalent in late and post-term births and was the only factor, along with maternal smoking, that was associated with adverse neonatal outcomes. Since nulliparity, CCA and GA beyond term are associated with HCA, this should alert the clinician and elicit continuous intrapartum monitoring for timely intervention.

**Supplementary Information:**

The online version contains supplementary material available at 10.1186/s12884-024-06392-4.

## Introduction

Pregnancy complications and adverse neonatal outcomes, such as stillbirth and neonatal death, become increasingly prevalent after 40 weeks’ gestation [1, 2]. To address the increased risk, a Swedish multicentre randomised trial concluded that induction of labour instead of expectant management should be recommended at 41 weeks, [3] and this was supported by another study in the Netherlands [4]. However, this study also demonstrated that induction of labour after 41 weeks increased the rates of adverse neonatal outcomes such as 5-minute Apgar score < 7, admission to the neonatal intensive care unit (NICU), and emergency caesarean section. With up to one in four pregnant women in the developed world having their labour induced [5], the high cost to health services and the impact on women and their families, there is an imperative need to better understand the factors that can identify high-risk pregnancies or women who would benefit from labour induction. Despite the acknowledged link between placental dysfunction and increased risk of chronic diseases later in life, [6] few studies have explored the connection between neonatal morbidity and underlying placental pathology. A recent paper by Nikkels et al. [7] compared placental findings in uneventful term deliveries with cases of NICU admission or perinatal death. They found more placental pathology in neonates with severe perinatal morbidity and mortality, which is in accordance with studies including placentas from births with adverse outcome only [8–10]. Throughout pregnancy, the placenta matures to meet the increased needs of the fetus for oxygen and nutrients, but the process of placental ageing and decreasing functional capacity beyond term is not well understood. Research has focused on biological markers, gene expression and ancillary techniques that can assess or measure placental function. Nonetheless, with the exception of markers for preeclampsia, no marker has been found useful in a clinical setting [11, 12]. Consequently, the primary aim of this study was to assess placental pathology according to gestational age (GA) at birth; early-term/term and late-term/post-term births that align with the local criteria for placental examination. A secondary aim was to explore potential differences between these groups in terms of clinical characteristics and the risk of adverse neonatal outcomes. We hypothesised that placental pathology and related neonatal morbidity would differ according to GA at birth.

## Materials and methods

### Participants and study design

**Fig. 1:**
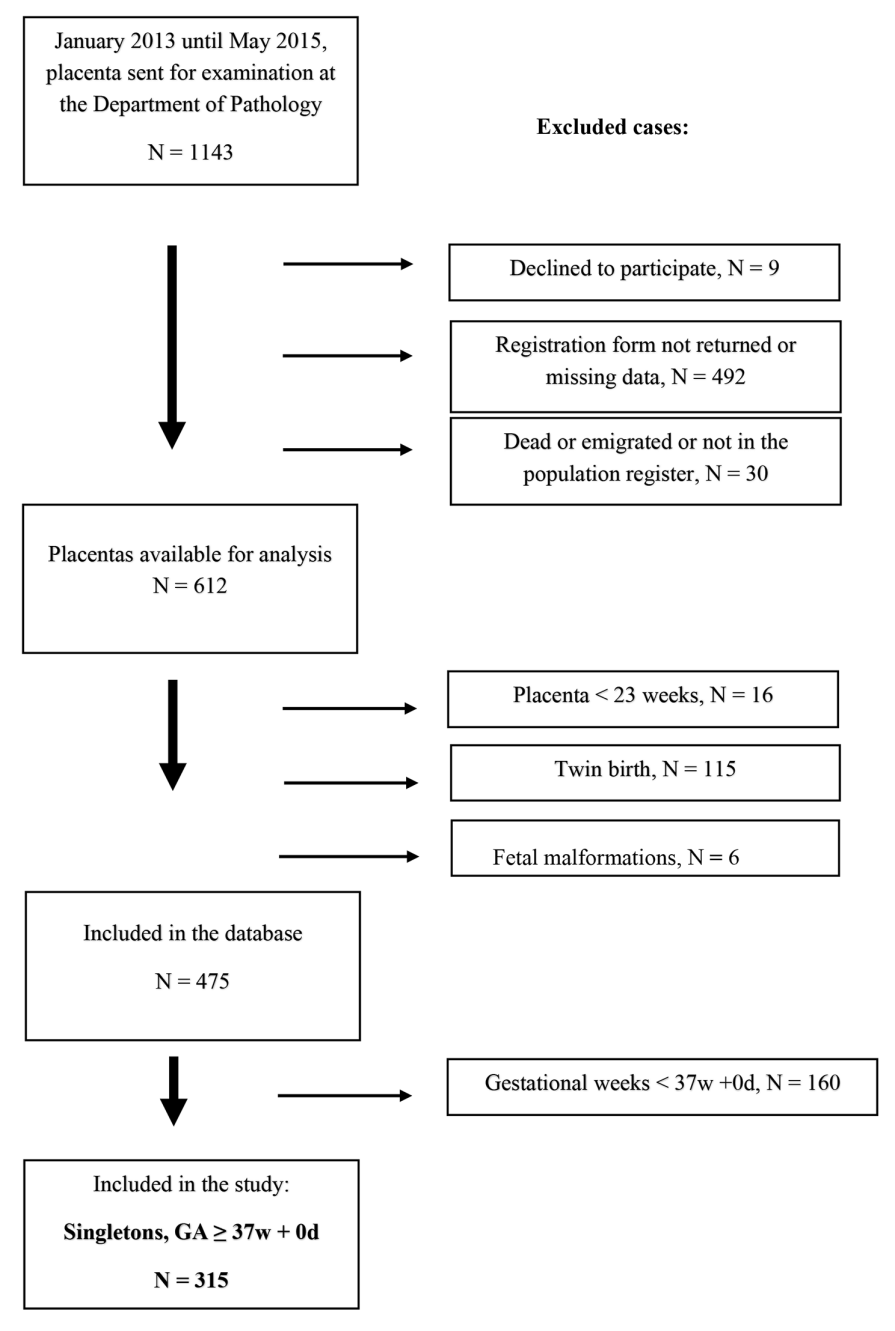
Flowchart for inclusion in a prospective observational study of 315 singleton term placentas meeting the criteria for referral to histopathologic examination

Women who delivered at Haukeland University Hospital *and* had a histological placental examination carried out according to local clinical routines (Supplementary S1) between January 2013 and May 2015 (*n* = 1143) were asked to enrol in our placental pathology database. The study required written informed consent, and eligible women were invited to participate. Of these, 492 (43.0%) did not complete the consent form, nine (0.8%) declined to participate, and 30 (2.6%) had emigrated from Norway (*n* = 29) or died (*n* = 1). Thus, a total of 612 (53.5%) placentas were included in the database. The present study includes births with GA > 36 week + 6 days without congenital malformations or syndromes, a total of 315 (Fig. 1).

The Regional Committees for Medical and Health Research Ethics approved the study (REK ID 2014/1926).

### Classification of placental histological findings

The placentas with the cord and membranes attached were grossly examined by the midwife attending the birth, rinsed in water, and fixated in a bucket with formalin. In the Pathology Department the placentas were measured in three dimensions and weighed without cord and membranes. Our department followed a standardised protocol, which included sampling of full-thickness sections from visually normal parenchyma at the inner two-thirds, sections from the cord insertion site, as well as two sections from the umbilical cord and membrane rolls, in agreement with the recommended guidelines [13]. Additionally, samples from macroscopically abnormal parenchyma were submitted. The histological evaluation was performed by a team (*N* = 5) of pathologists trained in perinatal pathology. Subsequently, two of those (EBB and KC) independently re-examined the slides to ensure consistency and accuracy of the findings.

Histologic chorioamnionitis (HCA) was defined as the presence of neutrophils in the chorionic plate or extraplacental membranes. Notably, a mild acute placental inflammation at term is considered to be part of the physiological process leading to parturition rather than an acute infection [10, 14–16]. Therefore, we dichotomised the maternal inflammatory response into two categories: *No inflammation/neutrophils in the subchorionic fibrin* (stage 0 or 1) versus *Neutrophils in the chorioamnion* (stage 2 or 3). Thus, only stage 2 or 3 were defined as presence of maternal inflammatory response.

A fetal inflammatory response (FIR) was defined as the presence of neutrophils in the wall of the chorionic vessels, the umbilical vein or arteries. This response was also divided into two groups of FIR: No and Yes.

Maternal vascular malperfusion (MVM) included a constellation of findings based on placental weight (weight < 10th centile), presence of villous infarctions or intervillous thrombi, and histological findings such as distal villous hypoplasia, accelerated villous maturation or decidual arteriopathy.

Fetal vascular malperfusion (FVM) was defined as thrombi (occlusive or non-occlusive) in large fetal vessels of the placenta, including umbilical, chorionic plate, and stem villous vessels or foci of avascular villi.

Delayed villous maturation (DVM) was defined as the presence of immature-appearing villi with poor vasculosyncytial membrane formation and centrally placed capillaries. To be classified as DVM, this abnormality had to be observed in at least 30% of a full thickness section.

Villitis was defined as a lymphohistiocytic infiltrate involving the chorionic villi and intervillositis as a diffuse infiltration of the intervillous space by monocyte-macrophages. These findings were not graded and were combined in the analyses due to the small number of cases.

The variables MVM, FVM, DVM and villitis/intervillositis were dichotomised into two groups: No (absence of the finding) or Yes (presence of the specific finding).

### Clinical variables

According to guidelines, [17] preeclampsia was defined as newly onset hypertension after gestation week 20 (blood pressure ≥ 140 mmHg systolic and/or ≥ 90 mmHg diastolic) combined with proteinuria or other signs of organ dysfunction.

Clinical chorioamnionitis (CCA) was diagnosed in the presence of maternal fever (rectal temperature ≥ 38 °C) accompanied by at least two of the following: maternal tachycardia (> 100 beats/minute), fetal tachycardia (> 160 beats/minute in at least 10 min), maternal leukocytosis (> 15 × 10^9^/L), uterine tenderness, or malodourous amniotic fluid.

GA was estimated based on ultrasound biometry in 1st trimester or at 17–19 weeks of gestation (92.8%) or according to in vitro fertilization data (7.0%). We divided the participants into four groups according to GA at delivery: early-term (37 weeks + 0 days to 38 weeks + 6 days), term (39 weeks + 0 days to 40 weeks + 6 days), late-term (41 weeks + 0 days to 41 weeks + 6 days) and post-term birth (42 weeks + 0 days to 42 weeks + 2 days).

Delivery outcomes as GA at birth, birthweight, information about the delivery, Apgar score, pH in umbilical cord artery, and admission to NICU were collected from the clinical files. A 5-min Apgar score < 7, umbilical cord artery pH < 7.0, NICU admission, or perinatal death were considered as adverse neonatal outcomes. The composite adverse neonatal outcome variable was none vs. at least one adverse outcome.

### Statistics

The chi-square or Fisher’s exact test was used for comparison of categorical variables, presented as proportions. For continuous variables, Student’s T-test or Mann-Whitney’s.

U-test was used, as appropriate, and presented as means with standard deviations. The adverse outcome measures were binary; therefore, a logistic regression model was used to estimate the odds ratio (OR) and 95% confidence intervals (CIs). Independent variables were included in the multivariate analysis if *p* < 0.2 in the unadjusted analysis, and only significant variables were included in the final model. P-values ≤ 0.05 were considered significant. The same procedure was used to explore associations between HCA and maternal and pregnancy characteristics. All analyses were performed using SPSS Statistics for Windows, version 26.0 (IBM Corp, New York, USA).

## Results

A total of 315 individual cases were included in the study (Fig. 1), among these 13 (4.1%) stillbirths. Table 1 presents the characteristics of the mothers, newborns, and placentas according to GA at birth. Delivery by emergency caesarean occurred more often in the late-term and post-term group compared to the early-term and term group (*p* = 0.0047). In addition, in late-term/post-term births, HCA and FIR were more common compared to early-term/term births (*p* = 0.004 and *p* = 0.001, respectively). This was accompanied by a higher rate of CCA (*p* = 0.023) and NICU transfer (*p* = 0.037). In contrast, MVM and preeclampsia were more common in early-term/term births, but the difference was not significant. A higher rate of small for gestational age neonates was also seen in the early-term/term group.

**Table 1:** Maternal, pregnancy and placental characteristics according to gestational age in a prospective observational study of 315 singleton term placentas meeting the criteria for referral to histopathologic examination

	Early-term	Term	Late-term	Post-term	
	GA 37^0^-38^6^	GA 39^0^-40^6^	GA 41^0^-41^6^	GA 42^0 –^ 42^2^	
**Maternal and pregnancy characteristics**	*n* = 118	*n* = 130	*n* = 55	*n* = 12	**P-value** ^a^
Maternal country of origin, *n/N (%)*					0.432
Norway	104/118 (88.1)	119/130 (91.5)	46/55 (83.6)	11/12 (91.7)	
Other	14/118 (11.9)	11/130 (8.5)	9/55 (16.4)	1/12 (8.3)	
Age (years), *mean (SD)*	31.4 (5.0)	31.2 (5.2)	31.6 (4.6)	30.4 (4.0)	
BMI (kg/m²), *mean (SD)*	24.0 (4.4)	24.5 (5.3)	24.8 (5.0)	26.7 (4.4)	
Smoking, *n/N (%)*	5/117 (4.3)	4/129 (3.1)	2/55 (3.6)	1/12 (8.3)	0.639
Para 0, *n/N (%)*	57/118 (48.3)	86/130 (66.2)	35/55 (63.6)	8/12 (66.7)	**0.029**
Preeclampsia^b^, *n/N (%)*	26/118 (22.0)	32/130 (24.6)	7/55 (12.7)	2/12 (16.7)	0.339
Severe preeclampsia^c^, *n/N (%)*	12/118 (10.2)	12/130 (9.2)	1/55 (1.8)	0/12 (0.0)	0.195
Clinical chorioamnionitis, *n/N (%)*	3/118 (2.5)	8/130 (6.2)	4/55 (7.3)	3/12 (25.0)	**0.023**
Pregestational diabetes, *n/N (%)*	8/118 (6.8)	5/130 (3.8)	0/55 (0.0)	0/12 (0.0)	0.206
Gestational diabetes, *n/N (%)*	8/118 (6.8)	8/130 (6.2)	3/55 (5.5)	1/12 (8.3)	0.922
PROM, *n/N (%)*	7/110 (6.4)	5/124 (4.0)	1/52 (1.9)	0/12 (0.0)	0.679
Onset of labor, *n/N (%)*					**< 0.001**
Spontaneous	45/118 (38.1)	79/130 (60.8)	24/55 (43.6)	2/12 (16.7)	
Induced	63/118 (53.4)	51/130 (39.2)	31/55 (56.4)	9/12 (75.0)	
Elective Caesarean	10/118 (8.5)	0/130 (0.0)	0/55 (0.0)	1/12 (8.3)	
Mode of delivery, *n/N (%)*					**0.047** ^**1**^
Spontaneous vaginal	52/118 (44.1)	54/130 (41.5)	18/55 (32.7)	3/12 (25.0)	
Forceps	19/118 (16.1)	18/130 (13.8)	4/55 (7.3)	2/12 (16.7)	
Vacuum	3/118 (2.5)	12/130 (9.2)	7/55 (12.7)	1/12 (8.3)	
Elective Cesarean	10/118 (8.5)	1/130 (0.8)	0/55 (0.0)	1/12 (8.3)	
Emergency Cesarean	34/118 (28.8)	45/130 (34.6)	26/55 (47.3)	5/12 (41.7)	
**Neonatal characteristics**					
Birthweight (g), *mean (SD)*	2830 (530)	3302 (555)	3646 (594)	3689 (567)	
Birth length (cm), *mean (SD)*	47.8 (2.7)	50.1 (2.3)	51.4 (2.1)	51.0 (1.4)	
Head circumference (cm), *mean (SD*)	33.7 (1.4)	34.5 (1.5)	35.7 (1.6)	35.8 (1.5)	
Small for gestational age^d^, *n/N (%)*	57/118 (48.3)	41/130 (31.5)	10/55 (18.2)	1/12 (8.3)	**< 0.001**
Large for gestational age^e^, *n/N (%)*	6/118 (5.1)	7/130 (5.4)	5/55 (9.1)	1/12 (8.3)	0.544
5-min Apgar score < 7, *n/N (%)*	13/118 (11.0)	15/130 (11.5)	2/55 (3.6)	2/12 (16.7)	0.231
Umbilical artery pH < 7.0, *n/N (%)*	4/94 (4.3)	7/106 (6.6)	2/48 (4.2)	2/9 (22.2)	0.195
NICU transfer, *n/N (%)*	28/118 (23.7)	21/130 (16.2)	14/55 (25.5)	6/12 (50.0)	**0.037**
Stillbirth, *n/N (%)*	5/118 (4.2)	8/130 (6.2)	0/55 (0.0)	0/12 (0.0)	0.270
Composite adverse outcome^f^, *n/N (%)*	37/118 (31.4)	33/130 (25.4)	14/55 (25.5)	6/12 (50.0)	0.256
**Placental and cord characteristics**					
Trimmed weight (g), *mean (SD)*	441 (121)	494 (112)	551 (137)	513 (104)	
Marginal/velamentous cord insertion, *n/N (%)*	8/118 (6.8)	16/130 (12.3)	3/55 (5.5)	1/12 (8.3)	0.392
Coiling index					0.283
Hypocoiled (< 1 coil/10 cm)	1/115 (0.9)	2/123 (1.6)	1/55 (1.8)	0/12 (0.0)	
Hypercoiled (> 3 coils/10 cm)	34/115 (29.6)	41/123 (33.3)	9/55 (16.4)	4/12 (33.3)	
**Microscopic diagnosis**					
No pathology^g^, n/N (%)	13/118 (11.0)	12/130 (9.2)	7/55 (12.7)	1/12 (8.3)	0.907
Histologic chorioamnionitis, *n/N (%)*					
Maternal inflammatory response^h^	9/118 (7.6)	21/130 (16.2)	11/55 (20.0)	5/12 (41.7)	**0.004**
Fetal inflammatory response^i^	10/118 (8.5)	27/130 (20.8)	14/55 (25.5)	5/12 (41.7)	**0.001**
Maternal vascular malperfusion	61/118 (51.7)	59/130 (45.4)	17/55 (30.9)	4/12 (33.3)	0.064
Fetal vascular malperfusion	11/118 (9.3)	11/130 (8.5)	3/55 (5.5)	0/12 (0.0)	0.772
Delayed villous maturation	20/118 (16.9)	27/130 (20.8)	7/55 (12.7)	1/12 (8.3)	0.554
Villitis/intervillositis	13/118 (11.0)	8/130 (6.2)	2/55 (3.6)	1/12 (8.3)	0.755
Two or more abnormalities	38/118 (32.2)	46/130 (35.4)	9/55 (16.4)	5/12 (41.7)	**0.047**

*Abbreviations*: GA = gestational age; SD = standard deviation; BMI = body mass index; PROM = prelabour rupture of membranes; NICU = neonatal intensive care unit; HELLP = hemolysis, elevated liver enzymes, low platelets

^a^Chi-square or Fisher’s exact test, ^b^Including four cases with hypertension in pregnancy, ^c^Including HELLP syndrome and eclampsia, ^d^Birthweight < 5 percentile, ^e^Birthweight > 90 percentile, ^f^Including any of the following: 5-min Apgar score < 7, umbilical artery pH < 7.0, NICU transfer, stillbirth, ^g^Including cases with Maternal inflammatory response stage 1 if no other placental pathology present, ^h^Maternal inflammatory response dichotomised into *no response/stage 1* versus *stage 2 or 3* (0/1), ^i^Fetal inflammatory response dichotomised into *no response* versus *any stage* (0/1)

^1^Dichotomised, early-term/term versus late-term/post-term

When the cases were stratified based on the presence of adverse neonatal outcome, HCA (including both the maternal and fetal inflammatory response) was more common in the adverse outcome group (22.2% vs. 11.6%, and 24.4% vs. 15.1%, respectively, Table 2). Additionally, the rate of smoking was higher in the adverse outcome group (8.9% versus 1.8%).

**Table 2:** Maternal, pregnancy and placental characteristics in neonates with and without adverse outcomes in a prospective observational study of 315 singleton term placentas meeting the criteria for referral to histopathologic examination

	Non-adverse outcome	Adverse outcome^a^	
**Maternal and pregnancy characteristics**	*n* = 225	*n* = 90	**P-value** ^**b**^
Maternal country of origin, *n/N (%)*			0.691
Norway	201/225 (89.3)	79/90 (87.8)	
Other	24/225 (10.7)	11/90 (12.2)	
Age (years), *mean (SD)*	31.3 (4.8)	31.3 (5.4)	0.995
BMI (kg/m²), *mean (SD)*	24.3 (4.6)	24.9 (5.7)	0.604^c^
Smoking, *n/N (%)*	4/223 (1.8)	8/90 (8.9)	**0.006**
Para 0, *n/N (%)*	130/225 (57.8)	56/90 (62.2)	0.469
Preeclampsia, *n/N (%)*	49/225 (21.8)	18/90 (20.0)	0.728
Severe preeclampsia^d^, *n/N (%)*	19/225 (8.4)	6/90 (6.7)	0.598
Clinical chorioamnionitis, *n/N (%)*	10/225 (4.4)	8/90 (8.9)	0.125
Pregestational diabetes, *n/N (%)*	10/225 (4.4)	3/90 (3.3)	0.764
Gestational diabetes, *n/N (%)*	13/225 (5.8)	7/90 (7.8)	0.511
PROM, *n/N (%)*	12/218 (5.5)	1/80 (1.3)	0.197
Onset of labor, *n/N (%)*			0.391
Spontaneous	110/225 (48.9)	40/90 (44.4)	
Induced	109/225 (48.4)	45/90 (50.0)	
Elective Caesarean	6/225 (2.7)	5/90 (5.6)	
Mode of delivery, *n/N (%)*			0.139
Spontaneous vaginal	99/225 (44.0)	28/90 (31.1)	
Forceps	31/225 (13.8)	12/90 (13.3)	
Vacuum	15/225 (6.7)	8/90 (8.9)	
Elective Cesarean	6/225 (2.7)	6/90 (6.7)	
Emergency Cesarean	74/225 (32.9)	36/90 (40.0)	
**Neonatal characteristics**			
Gestational age at delivery (weeks + days), n/N (%)			0.256
37 + 0 to 38 + 6	81/225 (36.0)	37/90 (41.1)	
39 + 0 to 40 + 6	97/225 (43.1)	33/90 (36.7)	
41 + 0 to 41 + 6	41/225 (18.2)	14/90 (15.6)	
42 + 0 to 42 + 2	6/225 (2.7)	6/90 (6.7)	
Birthweight (g), *mean (SD)*	3161 (630)	3298 (640)	0.085
Birth length (cm), *mean (SD)*	49.4 (2.6)	50.0 (3.1)	0.080
Head circumference (cm), *mean (SD)*	34.4 (1.6)	34.7 (1.9)	0.207
Small for gestational age^e^, *n/N (%)*	58/225 (25.8)	15/90 (16.7)	0.083
Large for gestational age^f^, *n/N (%)*	15/225 (6.7)	4/90 (4.4)	0.454
**Placental characteristics**			
Trimmed weight (g), *mean (SD)*	479 (124)	498 (126)	0.233
Marginal or velamentous cord insertion, *n/N (%)*	21/225 (9.3)	7/90 (7.8)	0.911
Coiling index			1.00
Hypocoiled (< 1 coil/10 cm)	3/225 (1.3)	1/80 (1.3)	
Hypercoiled (> 3 coils/10 cm)	65/225 (28.9)	23/80 (28.7)	
**Histology**			
No pathology^g^, *n/N (%)*	28/225 (12.4)	5/90 (5.6)	0.071
Histologic chorioamnionitis, *n/N (%)*			
Maternal inflammatory response^h^	26/225 (11.6)	20/90 (22.2)	**0.015**
Fetal inflammatory response^i^	34/225 (15.1)	22/90 (24.4)	**0.050**
Maternal vascular malperfusion	107/225 (47.6)	39/90 (43.3)	0.497
Fetal vascular malperfusion	14/225 (6.2)	10/90 (11.1)	0.140
Delayed villous maturation	37/225 (16.4)	18/90 (20.0)	0.453
Villitis/intervillositis	16/225 (7.1)	8/90 (8.9)	0.631
Two or more abnormalities	64/225 (28.4)	34/90 (37.8)	0.106

*Abbreviations*: SD = standard deviation; BMI = body mass index; PROM = prelabour rupture of membranes; NICU = neonatal intensive care unit; HELLP = hemolysis, elevated liver enzymes, low platelets

^a^Including any of the following: 5-min Apgar score < 7, umbilical artery pH < 7.0, NICU transfer, stillbirth, ^b^Chi-square or Fisher’s exact test, ^c^Mann-Whitney U-test, ^d^Including HELLP syndrome and eclampsia, ^e^Birthweight < 5 percentile, ^f^Birthweight > 90 percentile, ^g^Including cases with Maternal inflammatory response stage 1 if no other placental pathology present, ^h^Maternal inflammatory response dichotomised into *no response/stage 1* versus *stage 2 or 3* (0/1), ^i^Fetal inflammatory response dichotomised into *no response* versus *any stage* (0/1)

When exploring the association between adverse neonatal outcome and selected clinical and histological characteristics, we found that maternal smoking and HCA were associated with adverse neonatal outcome in the unadjusted logistic regression analyses (Table 3). This association persisted in the adjusted analysis (Table 3). We then explored the relationship between maternal and pregnancy characteristics and HCA. In the adjusted regression analysis, four factors were identified to influence the risk of HCA (Table 4). Nulliparous women had twice the risk of developing HCA (OR: 2.22, 95% CI: 1.01 to 4.86). The presence of CCA was associated with an almost sixfold increased risk of HCA (OR: 5.97, 95% CI: 1.99 to 17.91). There was also an association between HCA and emergency caesarean section and advanced gestational age (OR: 2.05, 95% CI: 1.01 to 4.15 and OR: 1.45, 95% CI: 1.13 to 1.87, respectively).

**Table 3:** Results from logistic regression analyses of composite adverse outcome^a^ according to maternal, pregnancy and histologic characteristics in a prospective observational study of 315 singleton term placentas meeting the criteria for referral to histopathologic examination

	Composite adverse outcome							
	**Unadjusted models**			**Adjusted model** ^b^				
**Characteristics**	**OR**	**95% CI**	**p-value**	**OR**	**(95% CI)**	**p-value**		
Age^c^ (years)	1.00	(0.95, 1.05)	0.994					
BMI ≥ 30 (kg/m²)	1.24	(0.56, 2.73)	0.599					
Smoking	**5.34**	**(1.57, 18.22)**	**0.005**	**5.10**	**(1.48, 17.60)**	**0.008**		
Primiparity	1.20	(0.73, 1.99)	0.467					
Gestation week^d^	0.98	(0.83, 1.16)	0.816					
Preeclampsia	0.90	(0.49, 1.65)	0.726					
Clinical chorioamnionitis	2.10	(0.80, 5.50)	0.140					
Pregestational diabetes	0.74	(0.20, 2.76)	0.648					
Gestational diabetes	1.38	(0.53, 3.57)	0.519					
PROM	0.22	(0.03, 1.70)	0.074					
Histologic chorioamnionitis^e^	**2.19**	**(1.15, 4.16)**	**0.019**	**2.10**	**(1.09, 4.04)**	**0.029**		
Fetal inflammatory response^f^	1.82	(0.99, 3.23)	0.056					
Maternal vascular malperfusion	0.71	(0.43, 1.18)	0.183					
Fetal vascular malperfusion	2.10	(0.91, 4.82)	0.086					
Delayed villous maturation	1.27	(0.68, 2.37)	0.457					

*Abbreviations*: OR = odds ratio; CI = confidence interval; PROM = prelabour rupture of membranes; HELLP = hemolysis, elevated liver enzymes, low platelets; NICU = neonatal intensive care unit

^a^Including any of the following: 5-min Apgar score < 7, umbilical artery pH < 7.0, NICU transfer, stillbirth; ^b^Includes all characteristics significant at 5% level in the unadjusted models, ^c,d^As continuous variables, ^e^Dichotomised into *no response/maternal stage 1* versus *maternal stage 2 or 3*, ^f^Dichotomised into *no response* versus *any stage* (0/1)

**Table 4:** Results from logistic regression analyses of histologic chorioamnionitis, maternal inflammatory response^a^ according to maternal and pregnancy characteristics in a prospective observational study of 315 singleton term placentas meeting the criteria for referral to histopathologic examination

	Histologic chorioamnionitis, maternal inflammatory response						
	**Unadjusted models**			**Adjusted model** ^b^			
**Characteristics**	**OR**	**95% CI**	**p-value**	**OR**	**95% CI**	**p-value**	
Mother not born in Norway	1.24	(0.49, 3.18)	0.658				
Maternal age ≥ 35 years	0.72	(0.34, 1.52)	0.377				
BMI ≥ 30 (kg/m²)	0.92	(0.33, 2.53)	0.871				
Smoking	2.00	(0.52, 7.68)	0.340				
Primiparity	**2.86**	**(1.36, 5.99)**	**0.003**	**2.22**	**(1.01, 4.86)**	**0.039**	
Preeclampsia	0.51	(0.21, 1.26)	0.122				
Severe preeclampsia^c^	0.23	(0.03, 1.72)	0.073				
Clinical chorioamnionitis	**11.76**	**(4.28, 32.33)**	**< 0.001**	**5.97**	**(1.99, 17.91)**	**0.001**	
Pregestational diabetes	0.48	(0.06, 3.75)	0.436				
Gestational diabetes	2.07	(0.71, 5.99)	0.206				
PROM	1.89	(0.50, 7.18)	0.374				
Emergency section	**3.19**	**(1.68, 6.05)**	**< 0.001**	**2.05**	**(1.01, 4.15)**	**0.049**	
SGA < 5 percentile	0.55	(0.24, 1.29)	0.150				
LGA > 90 percentile	1.61	(0.51, 5.09)	0.434				
Male sex	1.35	(0.72, 2.54)	0.352				
Gestation week^d^	**1.60**	**(1.27, 2.03)**	**< 0.001**	**1.45**	**(1.13, 1.87)**	**0.003**	

*Abbreviations*: OR = odds ratio; CI = confidence interval; BMI = body mass index; PROM = prelabour rupture of membranes; SGA = small for gestational age; LGA = large for gestational age; HELLP = hemolysis, elevated liver enzymes, low platelets

^a^Dichotomised into *no response/maternal stage 1* versus *maternal stage 2 or 3*, ^b^Includes all characteristics significant at 5% level in the unadjusted models, ^c^Including HELLP syndrome and eclampsia, ^d^As continuous variable

## Discussion

In this study of births from the early-term to the post-term period, we found that HCA was the most important factor associated with adverse neonatal outcome. Nulliparity, CCA and increasing GA all carried an increased risk of HCA.

Although there is agreement in guidelines to recommend induction of labour in post-term pregnancies, some argue for routine induction during the late-term or term period [18]. However, a large, randomised, multicentre trial (ARRIVE) on low-risk nulliparous women found that induction of labour at 39 weeks reduced the incidence of caesarean section but did not significantly lower the frequency of a composite adverse perinatal outcome [19]. A systematic review found that inducing labour at 39 weeks was associated with improved neonatal outcomes in both in nulliparous and primiparous women. However, in nulliparous women, induction of labour was associated with shoulder dystocia [20]. The external validity of these studies [19, 20] for the Norwegian practice is questionable since intervention rates during spontaneous and induced labour are substantially lower [21, 22]. There is some evidence that induction of labour may reduce perinatal death and morbidity [23]. This could explain the change in clinical practice with increased rates of inductions, but there is a trade-off against adverse outcomes due to interventions [24]. Therefore, it is important to identify risk factors of adverse outcome in order to differentiate care. Likewise, identifying predictors of induction success is important, and obstetric background factors such as maternal age and weight, insulin-dependent diabetes and obstetric history are considered. In a nationwide study from Denmark, maternal fever was significantly more common in the late-term group (GA 41 weeks + 4 days to 42 weeks + 0 days) than in the term group (GA 41 weeks + 0 days to 41 weeks + 3 days), which was also associated with an increased risk of neonatal morbidity [25]. Less attention has been paid to the placenta, although placental function is a major determinant for acute and unexpected adverse outcomes [26]. Placental function changes during pregnancy and may adapt according to fetal and maternal factors such as obesity, [27] maternal glucose metabolism and cardiovascular function [28]. It has taken a long time to reach consensus on the nomenclature of placental findings due to a shortage of pathologists with the necessary training and a lack of understanding of placental pathophysiology among clinicians [26]. As a result, placental reports are often delayed, which may have contributed to a lack of interest in placentas role in determining the optimal timing of induction in the next pregnancy [29]. Rather than finding associations between placental pathology and maternal and fetal factors, the focus has been on biological markers and ancillary techniques that can assess or measure placental function before labour. Although term HCA is considered a heterogeneous condition and often does not correspond to a clinical presentation, it is associated with adverse neonatal outcomes [30]. HCA has been found common in perinatal stroke [31] and associated with early onset sepsis in term births [32]. Interestingly, in a study by Roberts et al. that included only low-risk pregnancies, almost all HCA at term were non-infectious [33]. Milder inflammatory lesions are often seen in placentas with normal outcomes at term, and awareness should be raised when comparing low- and high-risk pregnancies if grading has not been taken into account [10]. 

In a study by Stormdal Bring et al. [34] umbilical cord complications and infections (defined as HCA) were more frequent in term (GA 37 weeks + 0 days to 40 weeks + 6 days) and post-term (GA ≥ 41 weeks + 0 days) stillbirths than in preterm stillbirths (GA ≤ 37 weeks + 0 days), corroborating our findings. The study by Nikkels et al. [7] confirmed that HCA was more frequent in stillbirths (antepartum and intrapartum) and neonatal deaths and in those admitted to NICU. In line with their findings, we also found that less frequent placental abnormalities such as fetal vascular malperfusion, delayed villous maturation and villitis of unknown etiology were more common in the adverse neonatal outcome group, but the number of events in our study was too small to examine this. Unlike their study, [7] which compared the outcome of uncomplicated pregnancies and births with groups of stillbirths, neonatal deaths and NICU survivors, we only included cases that met local clinical guidelines for histologic examination of the placenta. Therefore, we do not know the incidence of HCA in uncomplicated pregnancies and births in our region. On the other hand, in clinical practice it is not appropriate to send all placentas for histopathological examination, as most placentas from low-risk births have mild inflammatory or vascular lesions only and rarely provide additional significant information [10, 35]. 

Our study showed that HCA was inversely related to parity. This is in agreement with other reports [36]. Early HCA may represent the inflammatory process of labour itself and does not necessarily have an infectious etiology [15, 37]. As nulliparity is associated with longer duration of labour, the finding of early HCA in this group has been suggested to be a subclinical process [10]. We found that the subgroup with FIR, which is considered a higher burden lesion, was also more common in nulliparas (data not shown). Regardless of the cause of HCA, our results indicate that nulliparity should be considered as a risk factor for both HCA and FIR in term births.

Our results are further in line with previous studies that have found smoking to be a significant maternal factor associated with adverse neonatal outcomes [38, 39]. However, this association may be due to the confounding effect of smoking with other known causes of adverse outcomes, such as socioeconomic status and psychosocial stress [40]. On the other hand, the adverse effects of nicotine exposure on placental development are well known [41–43]. A recent study suggests that placental injury may induce a sterile inflammation through a shift in the pro- versus anti-inflammatory balance mediated by damage associated molecular patterns (DAMP) [44]. This is consistent with several other reports concluding that intrauterine inflammation can be triggered by many factors and supports theories about the complex relationship between intrauterine inflammation and infection [45]. 

One of the major challenges for obstetricians is to identify the group of term pregnancies that would benefit from induction of labour rather than expectant management at around 41 weeks. We found that neither preeclampsia nor MVM had an effect on neonatal outcome compared to other high-risk conditions. This may be explained by the fact that preeclamptic mothers are often induced earlier (37- and 38-weeks’ gestation). In a report by Lisonkova et al. [46] early-onset preeclampsia, but not late-onset preeclampsia, was associated with a high risk of fetal death. However, they also found that the rate of severe neonatal morbidity and death was higher in the group with late-onset preeclampsia compared with those without preeclampsia. Our findings are consistent with a report that concluded that late-onset preeclampsia (after 34 weeks’ GA) does not have a serious impact on neonatal complications, [47] although this was a small study involving only 77 newborns. Our results are further in line with a report by Levy et al., including placentas from preeclampsia cases routinely sent for assessment, that advanced GA and HCA were independently associated with adverse outcomes [48]. 

A strength of this study is that it is population-based and includes detailed maternal and neonatal characteristics. The placentas were investigated by perinatal pathologists according to international consensus criteria, and the diagnosis further strengthened by a second look. Similar to other perinatal studies, a composite variable was used to predict adverse outcomes. However, the use of a composite variable has its limitations as it often combines neonatal transition problems related to both the condition (such as placental insufficiency) and its treatment (such as relatively premature delivery) [49]. Additionally, there is a lack of information regarding the time needed to induce labour and the methods used. The pre-requisite of a clinical indication for placental investigation, and thus recruitment to the study, introduces a selection bias towards a high-risk cohort, which limits comparison and prediction statistics to be performed within this cohort of different maternal and fetal high-risk conditions. Comparison with a control group of uncomplicated pregnancies and births may have identified additional placental pathology associated with adverse outcomes.

## Conclusion

We found that in a cohort of term and late-term neonates, HCA and smoking during pregnancy were risk factors associated with adverse neonatal outcome. HCA was more common in the late-term group and was associated with nulliparity, CCA and advanced GA, which should increase awareness and surveillance during labour and delivery.

### Electronic supplementary material

Below is the link to the electronic supplementary material.

Supplementary Material 1

Supplementary Material 2

Supplementary Material 3

## Data Availability

The data that support the findings of this study are not available due to reasons of sensitivity and are available from the corresponding author upon reasonable request. Data are in controlled access data storage at Haukeland University Hospital.

## Declarations

It was approved by the regional committee for Medical Research Ethics Western Norway, Bergen University Hospital (REK ID 2014/1926).

## Abbreviations

GA: gestational age
HCA: histologic chorioamnionitis
CCA: clinical chorioamnionitis
NICU: neonatal intensive care unit
FIR: fetal inflammatory response
MVM: maternal vascular malperfusion
FVM: fetal vascular malperfusion
DVM: delayed villous maturation
