# Supplementary material for: Placental pathology and neonatal morbidity: exploring the impact of gestational age at birth
Source: BMC Pregnancy Childbirth. 2024 Mar 14;24:201. doi: 10.1186/s12884-024-06392-4 (PMC10938777; doi:10.1186/s12884-024-06392-4)
Supplement: Supplementary file 1 — Supplementary Material 1 [file 12884_2024_6392_MOESM1_ESM.docx]

**Supplementary S1, local guidelines**

Indications for submission of the placenta to the pathology department:

| **Maternal indications** |  |
| --- | --- |
|  | Severe preeclampsia or hypertensive disorder before GW 37 |
|  | Diabetes mellitus before GW 37 |
|  | Suspected chorioamnionitis |
|  | Abruptio placentae |
|  | Prepregnant BMI > 40 kg/m^2^ |
|  | Recurrent bleeding during pregnancy |
| **Fetal indications** |  |
|  | Intrauterine/perinatal death |
|  | NICU transmission |
|  | Acidosis, umbilical artery pH < 7.0 |
|  | Oligohydramnios |
|  | Birth weight < 2500 g or birth weight < 5 percentile |
|  | GA < 32 weeks |
| **Placental indications** |  |
|  | Abnormal findings as tumours, cysts, infarcts, or haemorrhage |
|  | Incomplete attachment of membranes over a large area |
|  | Velamentous insertion site |
|  | Placental weight > 1000 g or < 400 g (at term) |
| **Clinical judgement** |  |

*Abbreviations*: GW = gestation week, BMI = body mass index, GA= gestational age
