# Supplementary material for: Placental pathology and neonatal morbidity: exploring the impact of gestational age at birth
Source: BMC Pregnancy Childbirth. 2024 Mar 14;24:201. doi: 10.1186/s12884-024-06392-4 (PMC10938777; doi:10.1186/s12884-024-06392-4)
Supplement: Supplementary file 2 — Supplementary Material 2 [file 12884_2024_6392_MOESM2_ESM.docx]

**Table 5** Indications for induction of labour

|  | **Early-term** |  | **Term** |  | **Late-term** |  | **Post-term** | **Total** |
| --- | --- | --- | --- | --- | --- | --- | --- | --- |
|  | **GA 37^0^-38^6^** |  | **GA 39^0^-40^6^** |  | **GA 41^0^-41^6^** |  | **GA 42^0 -^ 42^2^** |  |
| **Indications for induction of labour, n (%)** | **n = 63** |  | **n = 51** |  | **n = 31** |  | **n = 9** | **n = 154** |
| PIH^a^/Preeclampsia | 10 (15.9) |  | 13 (25.5) |  | 5 (16.1) |  | 0 (0.0) | 28 (18.2) |
| Diabetes | 2 (3.2) |  | 7 (13.7) |  | 0 (0.0) |  | 0 (0.0) | 9 (5.8) |
| IUGR^b^ | 26 (41.3) |  | 10 (19.6) |  | 2 (6.5) |  | 0 (0.0) | 38 (24.7) |
| Late- and post-term | 0 (0.0) |  | 0 (0.0) |  | 2 (6.5) |  | 7 (77.8) | 9 (5.8) |
| Prolonged rupture of membranes | 4 (6.3) |  | 4 (7.8) |  | 3 (9.7) |  | 0 (0.0) | 11 (7.1) |
| Oligohydramnios | 0 (0.0) |  | 3 (5.9) |  | 7 (22.6) |  | 1 (11.1) | 11 (7.1) |
| Reduced fetal movements | 0 (0.0) |  | 1 (2.0) |  | 1 (3.2) |  | 1 (11.1) | 3 (1.9) |
| IUFD^c^ | 2 (3.2) |  | 6 (11.8) |  | 0 (0.0) |  | 0 (0.0) | 8 (5.2) |
| Other | 19 (30.2) |  | 7 (13.7) |  | 11 (35.5) |  | 0 (0.0) | 37 (24.0) |

^a^Pregnancy-induced hypertension, ^b^Intrauterine growth restriction, ^c^Intrauterine fetal death
