# Supplementary material for: Placental pathology and neonatal morbidity: exploring the impact of gestational age at birth
Source: BMC Pregnancy Childbirth. 2024 Mar 14;24:201. doi: 10.1186/s12884-024-06392-4 (PMC10938777; doi:10.1186/s12884-024-06392-4)
Supplement: Supplementary file 3 — Supplementary Material 3 [file 12884_2024_6392_MOESM3_ESM.docx]

Table 6 Methods of induction

|  | **Early-term** |  | **Term** |  | **Late-term** |  | **Post-term** | **Total** |
| --- | --- | --- | --- | --- | --- | --- | --- | --- |
|  | **GA 37^0^-38^6^** |  | **GA 39^0^-40^6^** |  | **GA 41^0^-41^6^** |  | **GA 42^0 -^ 42^2^** |  |
| **Methods of induction** | **n (%)** |  | **n (%)** |  | **n (%)** |  | **n (%)** | **n (%)** |
|  | **56 (47.5)** |  | **80 (61.5)** |  | **24 (43.6)** |  | **3 (25.0)** | **163 (51.7)** |
| Oxytocin | 0 (0.0) |  | 1 (0.8) |  | 1 (1.8) |  | 0 (0.0) | 2 (0.6) |
| Amniotomy | 1 (0.8) |  | 4 (3.1) |  | 3 (5.5) |  | 1 (8.3) | 9 (2.9) |
| Balloon catheter | 39 (33.1) |  | 27 (20.8) |  | 23 (41.8) |  | 5 (41.7) | 94 (29.8) |
| Prostaglandin (Cytotec) | 22 (18.6) |  | 16 (12.3) |  | 4 (7.3) |  | 3 (25.0) | 45 (14.3) |
| Other | 0 (0.0) |  | 2 (1.5) |  | 0 (0.0) |  | 0 (0.0) | 2 (0.6) |
